# Comparison of the composition of bile acids in bile of patients with adenocarcinoma of the pancreas and benign disease

**DOI:** 10.1016/j.jsbmb.2017.10.011

**Published:** 2017-11

**Authors:** David O. Rees, Peter J. Crick, Gareth J. Jenkins, Yuqin Wang, William J. Griffiths, Tim H. Brown, Bilal Al-Sarireh

**Affiliations:** a Swansea University Medical School, ILS1 Building, Singleton Park, Swansea SA2 8PP, UK; b Department of Surgery, Morriston Hospital, Swansea, SA6 6NL, UK

**Keywords:** Pancreatic cancer, Liquid chromatography–mass spectrometry, Bile, Bile acids

## Abstract

Bile acids have been implicated in the development of gastrointestinal malignancies. Both the specific nature of individual bile acids and their concentration appear key factors in the carcinogenic potency of bile. Using liquid chromatography mass spectrometry (LC–MS) we performed quantitative profiling of bile extracted directly from the common bile duct in 30 patients (15 patients with pancreatic cancer and 15 patients with benign disease). Separation and detection of bile acids was performed using a 1.7 μm particle size reversed-phase C_18_ LC column at a flow rate of 200 μL/min with negative electrospray ionization MS. A significant difference (p = 0.018) was seen in the concentration of unconjugated cholic acid in the malignant group (0.643 mmol/L) compared to the benign group (0.022 mmol/L), with an overall significant difference (p = 0.04) seen in the level of total unconjugated bile acids in the malignant group (1.816 mmol/L) compared to the benign group (0.069 mmol/L). This finding may offer the possibility of both understanding the biology of cancer development in the pancreas, as well as offering a potential diagnostic avenue to explore. However, a larger study is necessary to confirm the alterations in bile acid profiles reported here and explore factors such as diet and microbial populations on the bile acid profiles of these patient groups.

## Highlights

• Bile acids and adenocarcinoma of the pancreas.
• Bile acids extracted directly from common bile duct of patients undergoing surgery.
• LC–MS analysis of bile acids.
• Significantly elevated levels of unconjugated cholic acid in patients with adenocarcinoma of the pancreas.

## Graphical abstract

**Figure:**
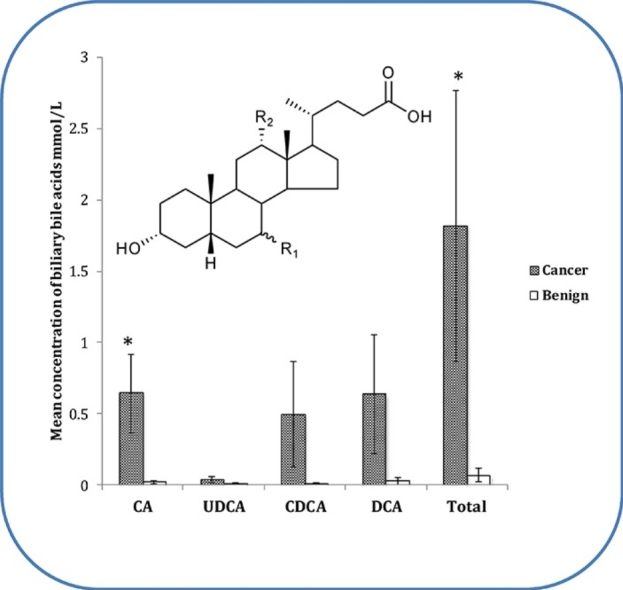

## 1 Introduction

In the United Kingdom 21 people are diagnosed with ductal adenocarcinoma of the pancreas each day, with only 3% of people surviving 5 years after diagnosis [1]. Currently, only surgery offers the possibility of cure [2]. A better understanding of the pathogenesis of this disease is required in order that prevention, early detection and effective treatments are realised. Bile acids are a normal component of the gastrointestinal (GI) tract, where they enable absorption of lipids, cholesterol and fat-soluble vitamins. Despite this, there is accumulating evidence that implicates bile acids in the development of GI malignancies including oesphageal, stomach and colon cancers [3], [4]. The majority of pancreatic cancers occur at the head of the pancreas, which is in close proximity to the bile duct raising the possibility that bile acids have a role in the pathogenesis of this cancer. In animal studies, where surgical procedures have been undertaken to alter the anatomy causing bile to reflux into the pancreatic duct, an increased incidence of pancreatic malignancy is seen [5].

In the human body, bile acid composition is regulated by synthesis, mostly in the liver, and through the enterohepatic circulation. So called “primary bile acids” (cholic acid, chenodeoxycholic acid) are conjugated to the amino acids glycine and taurine in the liver, prior to storage in the gallbladder and release first into the bile duct, and then duodenum. Greater than 95% of primary bile acids are reabsorbed in the terminal ileum. In the colon, primary bile acids are deconjugated and converted to so called “secondary bile acids” (e.g. deoxycholic acid, ursodeoxycholic acid and lithocholic acid) by bacterial flora. The secondary bile acids deoxycholic acid and ursodeoxycholic acid are partly absorbed in the colon and enter the enterohepatic circulation, whilst lithocholic acid is largely insoluble [6], [7]. The structure of bile acids allows them to act as detergents, disrupting the lipid bi-layer of cells, potentially allowing carcinogenic substances to enter the cell [4], [8]. Secondary bile acids have been specifically shown to have carcinogenic properties. Deoxycholic acid has been shown to generate reactive oxygen species which can cause apoptosis of cells [9]. Additionally, in animal studies a diet high in the primary bile acid cholic acid leads to an increased resistance to apoptosis of colon crypt cells [10]. Bernstein et al. hypothesize that this favours selection of apoptosis resistant cells, predisposing to the development of malignancy [3]. Nagathihalli et al. have shown deoxycholic acid to activate EGFR signaling, by cleavage of the protein amphiregulin, leading to cell proliferation [11]. Cyclooxygenase-2 (COX-2) and its enzymatic product prostaglandin E_2_ (PGE_2_) have been implicated in tumour formation [12], [13]. Tucker et al. demonstrated upregulation of COX-2 in human pancreatic cell lines when treated with chenodeoxycholic and deoxycholic acid, an increase in PGE_2_ was also observed [14].

Both the specific nature of individual bile acids and their concentration appear key factors in the carcinogenic potency of bile. Liquid chromatography-mass spectrometry (LC–MS) is an established technique to accurately characterise and measure individual bile acids in biological fluids [15]. The aim of this study is to analyse bile acid profiles using LC–MS, by extracting bile directly from the common bile duct of patients with pancreatic cancer and benign disease.

## 2 Materials and methods

### 2.1 Patients

The study was divided into two groups of patients attending for upper gastrointestinal surgery at Morriston Hospital, Swansea. The first group of patients had a diagnosis of cholecystitis, benign disease, and underwent cholecystectomy surgery. This group consisted of 15 patients, 12 female and 3 male. The second group of patients underwent pyloris preserving pancreaticoduodenectomy and were diagnosed with adenocarcinoma of the pancreas, after histopathological examination (Table 1). This group consisted of 15 patients, 6 female and 9 male. In both groups the bile sample was obtained by direct extraction from the common bile duct during surgery. In patients undergoing pyloris preserving pancreaticoduodenectomy pancreatic fluid was also collected directly from the pancreatic duct. All patients had been fasted from midnight. No special dietary instructions were given to either group. Prior to surgery, serum liver function tests (alkaline phosphatase (ALP), alkaline transferase (ALT) and bilirubin) of patients were also carried out.

**Table 1:** Demographic and histopathological characteristics of patients.

Disease	Cholecystitis	Adenocarcinoma of the pancreas
Sex (males/females)	3/12	9/6
Age (years)	50 (17–85)	62 (52–75)

Staging		
Adenocarcinoma pT1 N0	0	1
Adenocarcinoma pT2 N1	0	1
Adenocarcinoma pT3 N1	0	9
Adenocarcinoma pT3 N0	0	4

### 2.2 Ethical aspects

Informed consent for extraction, storage and analysis of biliary fluid was obtained from all 30 patients undergoing elective surgery, with ethical permission obtained from South West Wales Research and Ethics Committee (10/WMW02/34).

### 2.3 Reagents

Bile acid standards (cholic acid and deoxycholic acid) were obtained from Sigma Aldrich (Dorset, UK) and were at least 98% pure. The internal standard, 3α,12α-dihydroxy-23-*nor*-5β-cholanoic acid, was obtained from Steraloids (Newport, RI, USA). Other bile acid standards were from previous studies [15]. HPLC grade solvents and reagents were from Fisher Scientific (Loughborough, UK) or Sigma Aldrich.

### 2.4 Preparation of internal standards

Taurine conjugated 3α,12α-dihydroxy-23-*nor*-5β-cholanoic acid and glycine conjugated 3α,12α-dihydroxy-23-*nor*-5β-cholanoic acid were synthesized as described previously for use as internal standards [16]. LC–MS relative response factors were calculated for accurately prepared solutions of unconjugated, glycine conjugated and taurine conjugated bile acid standards.

### 2.5 Extraction for bile acid analysis

Bile (1 μL) was added to 1 mL of 50% HPLC grade methanol and heated at 60 °C for 30 min. The mixture was centrifuged at 14,000*g* for 30 min at 4 °C. The supernatant was removed and stored at −80 °C. 3α,12α-Dihydroxy-23-*nor*-5β-cholanoic acid as well as the corresponding glycine and taurine conjugates were added as internal standards. The resulting mixture was diluted by a factor of 10,000 prior to analysis by LC–MS.

### 2.6 LC–MS analysis on the LTQ-Orbitrap

Separation of bile acids was performed on an Ultimate 3000 HPLC System (Dionex, now Thermo Fisher, Hemel Hempstead, UK) using a Phenomonex Kinetex C_18_ column (50 × 2.1 mm, 1.7 μm particle size, Macclesfield, UK). Mobile phase A consisted of 33.3% methanol, 16.7% acetonitrile, 0.1% formic acid. Mobile phase B consisted of 63.3% methanol, 31.7% acetonitrile, 0.1% formic acid. The flow rate was 200 μL/min. The gradient started at 20% mobile phase B for 1 min before rising to 80% mobile phase B over the following 7 min. After a further 5 min, the gradient returned to 20% B over 6 s before re-equilibration for 3 min 54 s to give a total run time of 17 min. The eluent was directed to the atmospheric pressure ionization (API) source of an LTQ-Orbitrap Elite mass spectrometer (Thermo Fisher) operating in negative ion electrospray mode. 50 μL of the diluted sample was injected onto the column and a full mass spectrum was recorded in the Orbitrap over the *m*/*z* range 350–700 at 120,000 resolution (at *m*/*z* 400, FWHM definition). The Orbitrap was calibrated externally prior to each analytical session and the mass error was less than 5 ppm.

### 2.7 Statistical analysis

Analysis was performed using SPSS for Windows. No assumptions of normality were made and the non-parametric Mann Whitney *U*-test was used to compare malignant to benign groups. A p-value <0.05 was considered statistically significant.

## 3 Results

Bile from 30 patients was analysed. 15 patients had a diagnosis of cholecystitis, the remaining 15 patients had a diagnosis of adenocarcinoma of the pancreas (Table 1). Analysis of serum liver function tests showed no significant difference between the levels of ALP or bilirubin in patient serum when comparing the benign and malignant groups (Table 2).

**Table 2:** Serum biochemical parameters of patients, p < 0.05 is significant.

	Cholecystitis	Adenocarcinoma of the pancreas	p-value
Total serum bilirubin (μmol/L) (prior to surgery)	13.9 (3–50)	15.3 (4–95)	0.280
Alkaline phosphatase (ALP) (U/L) (prior to surgery)	146.9 (70–782)	108 (53–312)	0.497

Bile samples were analysed by LC–MS in the negative ion mode. The carboxylic, or sulphonic, acid moiety of bile acids results in the formation of intense [M-H]^−^ ions that are readily detected. To obtain quantitative data we added a commercially available unnatural truncated bile acid (3α,12α-dihydroxy-23-*nor*-5β-cholanoic acid) as an internal standard. This compound has a similar structure to deoxycholic acid but with one less methylene group in the side chain and therefore has similar physicochemical properties. In addition, we synthesized the corresponding glycine and taurine conjugates of 3α,12α-dihydroxy-23-*nor*-5β-cholanoic acid for use as internal standards.

**Fig. 1:**
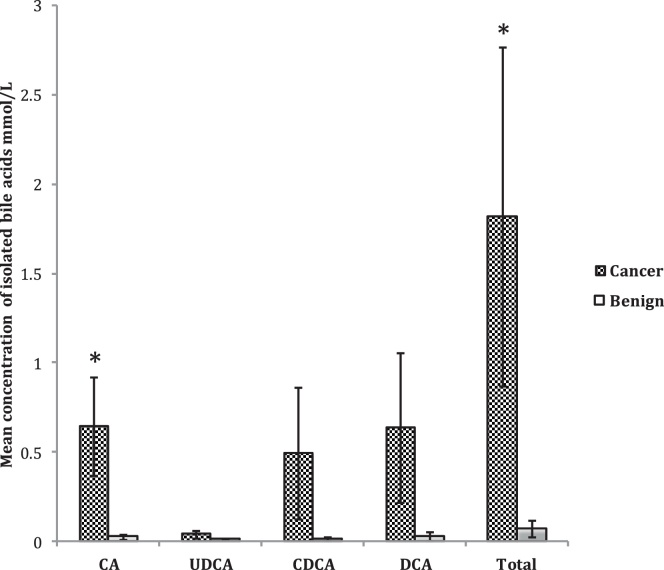
Comparison of biliary unconjugated bile acid concentrations (mmol/L) from benign and malignant samples. Standard error of the mean indicated by the error bars. CA, cholic acid, UDCA, urosodeoxycholic acid; CDCA, chenodeoxycholic acid; DCA, deoxycholic acid. *, p < 0.05.

**Fig. 2:**
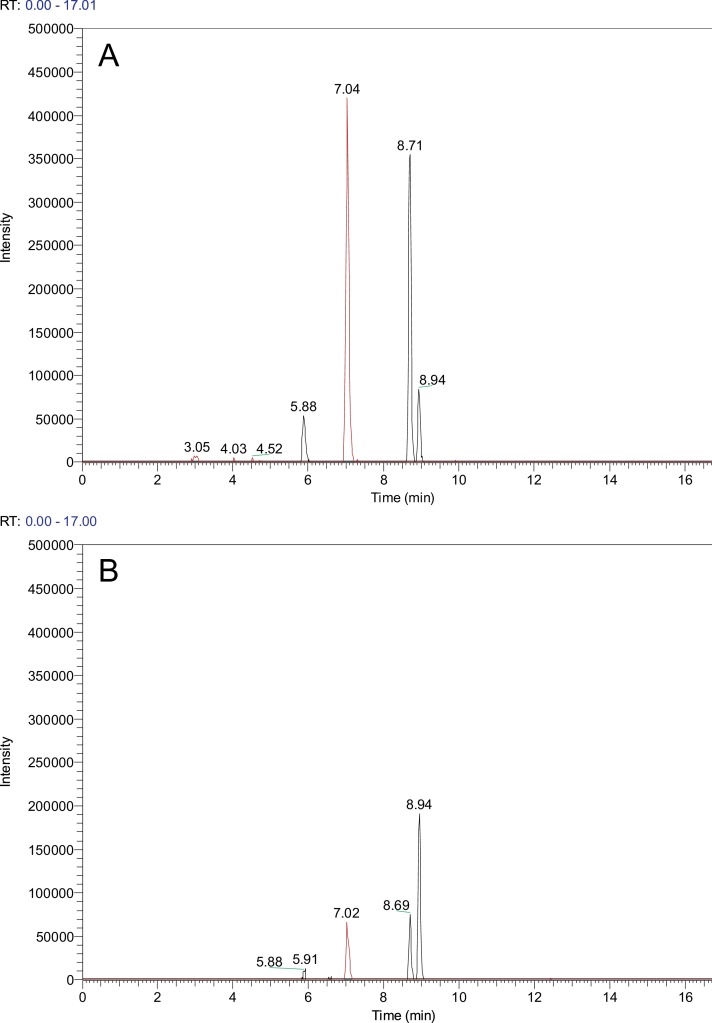
Reconstructed ion chromatograms (±5 ppm) showing [M−H]^−^ ions of ursodeoxycholic acid (*m*/*z* 391.2854, Rt 5.9 min), cholic acid (*m*/*z* 407.2803, Rt 7.0 min), chenodeoxycholic acid (*m*/*z* 391.2854, Rt 8.7) and deoxycholic acid (*m*/*z* 391.2854, Rt 8.9) from a patient with (A) adenocarcinoma and (B) with cholecystitis. The peaks corresponding to cholic acid are in red. Rt, retention time.

Individual and total bile acids in the benign group were compared to the malignant group (Fig. 1, Table 3). A trend towards a higher concentration of individual unconjugated bile acids was seen in the malignant group compared to the benign group (Fig. 2). A significant difference (p = 0.018) was seen in the concentration of unconjugated cholic acid in the malignant group (0.643 mmol/L) compared to the benign group (0.022 mmol/L), with an overall significant difference (p = 0.04) seen in the level of total unconjugated bile acids in the malignant group (1.816 mmol/L) compared to the benign group (0.069 mmol/L). No significant difference was seen when comparing the concentrations of the conjugated bile acids. Sulphate, glucuronide and glucoside derivatives of the bile acids were not detected in either benign or malignant samples. Bile acids were not detected in the pancreatic fluid collected.

**Table 3:** Comparison of concentrations of isolated bile acids in benign and malignant groups following LC–MS, p < 0.05 is significant.

Bile acid	Mean concentration (mmol/L)		Standard error of the mean (mmol/L)		p-value
	Benign	Malignant	Benign	Malignant	
Cholic acid	0.022	0.643	0.012	0.277	**0.018**
Ursodeoxycholic acid	0.010	0.038	0.003	0.019	0.289
Chenodeoxycholic acid	0.010	0.493	0.010	0.370	0.114
Deoxycholic acid	0.027	0.636	0.027	0.417	0.126
Lithocholic acid	0.000	0.000	N/A	N/A	N/A
Total unconjugated bile acids	0.069	1.816	0.047	0.950	**0.040**
Glychocholic acid	32.831	44.361	5.837	6.808	0.215
Glycoursodeoxycholic acid	3.177	3.206	1.192	1.387	0.741
Glycochenodeoxycholic acid	28.405	34.324	4.962	4.399	0.407
Glycodeoxycholic acid	27.169	29.101	7.127	4.740	0.453
Glycolithocholic acid	1.418	1.433	0.531	0.601	1
Total glycine conjugative bile acids	94.526	113.345	18.374	13.557	0.215
Taurocholic acid	12.718	20.127	2.719	6.454	0.342
Tauroursodeoxycholic acid	0.578	0.388	0.233	0.103	0.711
Taurochenodeoxycholic acid	12.809	14.979	3.788	3.866	0.430
Taurodeoxycholic acid	9.036	9.524	3.011	2.167	0.430
Taurolithocholic acid	0.465	0.566	0.133	0.182	0.968
Total taurine conjugated bile acids	35.935	45.883	9.368	12.354	0.384
Total bile acids	130.531	161.044	25.975	24.814	0.197

p-values shown in bold are considered statistically significant.

A limited bivariate correlation was performed between bile acid concentrations and serum biochemical tests (Table 4). In the malignant group, no significant correlation was seen between serum bilirubin and unconjugated cholic acid or between serum bilirubin and total unconjugated bile acid. Interestingly, a significant correlation was seen in the benign group between serum ALP and unconjugated cholic acid (p < 0.01) and serum ALP and total unconjugated bile acid (p < 0.01).

**Table 4:** Bivariate correlation between bile acid concentrations and biochemical test, p < 0.05 is significant.

		Bilirubin (μmol/L)	ALP (U/L)
Cholic acid (benign group)	Pearson correlation	−0.193	0.753
	p-value	0.490	0.001

Cholic acid (malignant group)	Pearson correlation	0.084	−0.025
	p-value	0.767	0.930

Total unconjugated bile acid (benign group)	Pearson correlation	−0.101	0.852
	p-value	0.720	0.00006

Total unconjugated bile acid (malignant group)	Pearson correlation	−0.126	−0.063
	p-value	0.655	0.823

## 4 Discussion

Previous studies have linked high physiological concentrations of bile acids and of specific bile acids to GI malignancies [3], [17]. In this study we compared the composition of bile from patients with cholecystitis and adenocarcinoma of the pancreas. Each sample was collected directly from the common bile duct during the patient’s planned surgery. There was general agreement between concentrations of bile acids measured with those previously reported [15]. A significant difference was found between the benign and malignant groups in the concentration of unconjugated cholic acid and in the total unconjugated bile acids (Fig. 1, Table 3). Higher concentrations of the other unconjugated bile acids were also seen in the malignant group compared to the benign, however these were not significant and may reflect the relatively low numbers of patients involved. These results are encouraging in terms of conducting a future analysis on a larger group of patients.

We report here results of a direct comparison between bile acid profiles of patients with adenocarcinoma at the head of the pancreas and benign disease. Wen et al. used nuclear magnetic resonance to compare bile from patients with biliary tract cancer and benign disease [18]. Applying orthogonal partial least square discriminant analysis (OPLS-DA), results showed good distinction between cancer and benign groups, the OPLS-DA model was found to be more sensitive and specific compared to conventional biomarkers. Both Xiao et al. and Zhang et al. have used LC–MS in an attempt to identify biomarkers in hepatocellular carcinoma (HCC) [19], [20]. Interestingly, Xiao et al. showed serum glycocholic acid (GCA) to be reduced in patients with HCC compared to those with liver cirrhosis, whilst Zhang et al. found GCA to be elevated in the urine of patients with HCC compared to healthy controls [19], [20]. Using high performance liquid chromatography (HPLC) Jusakul et al. compared biliary bile acid profiles taken from patients undergoing liver resection for malignant disease and benign biliary disease and showed the concentration of cholic acid to be significantly higher in cholangiocarcinoma and HCC patients compared to the benign group [21]. Jusakul et al. also demonstrated a significant correlation between serum bilirubin and total cholic acid, concluding that bile duct obstruction may promote bile acid synthesis in the liver, catalyzing malignant growth [21]. However, unlike Jusakul et al. we found no correlation between serum biochemistry and bile acids in the malignant group.

The biological mechanism to explain the differences in the bile acid profiles is uncertain. One explanation for the increased concentration of unconjugated bile acids extracted from the common bile duct (CBD) in the malignant group is the presence of bacteria in and around the duct producing hydroxylases in the CBD. Research conducted on CBD stones may offer an explanation. CBD stones prevent the flow of bile into the duodenum, leading to bile stasis which has been shown to cause bacterial growth [22]. As with a CBD stone, a tumour at the head of the pancreas can also cause bile duct obstruction.

No bile acids were detected upon analysis of pancreatic fluid, therefore the hypothesis that bile acids reflux to the pancreatic duct and lead to pancreatic inflammation has not been proven. In a review by Lerch et al., one argument against reflux is pressure in the pancreatic duct is generally quoted as being much higher than in the CBD [23]. Interestingly, a study by Csendes et al. agreed with this finding [24], however, the authors reported that one limitation of their study was the difference in the diameters of the pancreatic duct and CBD which could effect the pressures recorded when using the same size catheter probe. Csendes’ research highlights the difficulty in analysing physiological systems, and reflects the ongoing debate in the literature regarding the role of bile acids in pancreatic disease pathophysiology. Bile acids have been shown to have a direct affect on acinar cells initiating an inflammatory process, with membrane co-transporters for bile acids found on the luminal and basolateral surface of acinar cells [25]. Although all patients in this study were fasted overnight, one factor not taken into account was patient diet. High fat diets have been shown to increase the concentration of bile acid in faeces [26]. Epidemiological research has shown an increased incidence of colorectal cancer in populations with an increased faecal bile acid concentration [27], [28]. Deoxycholic acid has been implicated in the development of colorectal cancer [27], [29]. In our study, although an increased concentration of unconjugated deoxycholic acid was seen in the malignant group it was not significant.

Having found a significant difference in bile acid profiles in benign and malignant groups one clinical application from this study is the potential development of a biomarker. New biomarkers are integral to improving survival in pancreatic cancer through early diagnosis and in the monitoring of disease activity. Exploring whether the results found here could be replicated in serum samples would be therefore of great interest [30].

## 5 Conclusion

This study has shown a significant increase in both unconjugated cholic acid and total unconjugated bile acids when comparing patients with adenocarcinoma of the pancreas to those with benign disease. The mechanism by which this occurs is unclear. A drawback of this study is the relatively low samples numbers. A larger study is necessary to confirm the alterations in bile acid profiles, particularly exploring the trend towards higher concentrations seen in individual unconjugated bile acids from the malignant group. In addition, incorporating the influence of factors such as diet and microbial populations on the bile acid profiles in patients would be of benefit in analysing future results.
